# Supplementary material for: Complex‐centric proteome profiling by SEC‐SWATH‐MS
Source: Mol Syst Biol. 2019 Jan 14;15(1):e8438. doi: 10.15252/msb.20188438 (PMC6346213; doi:10.15252/msb.20188438)
Supplement: Supplementary file 8 — Dataset EV7 [file MSB-15-e8438-s008.zip › feature_plots_string/P12074.pdf]

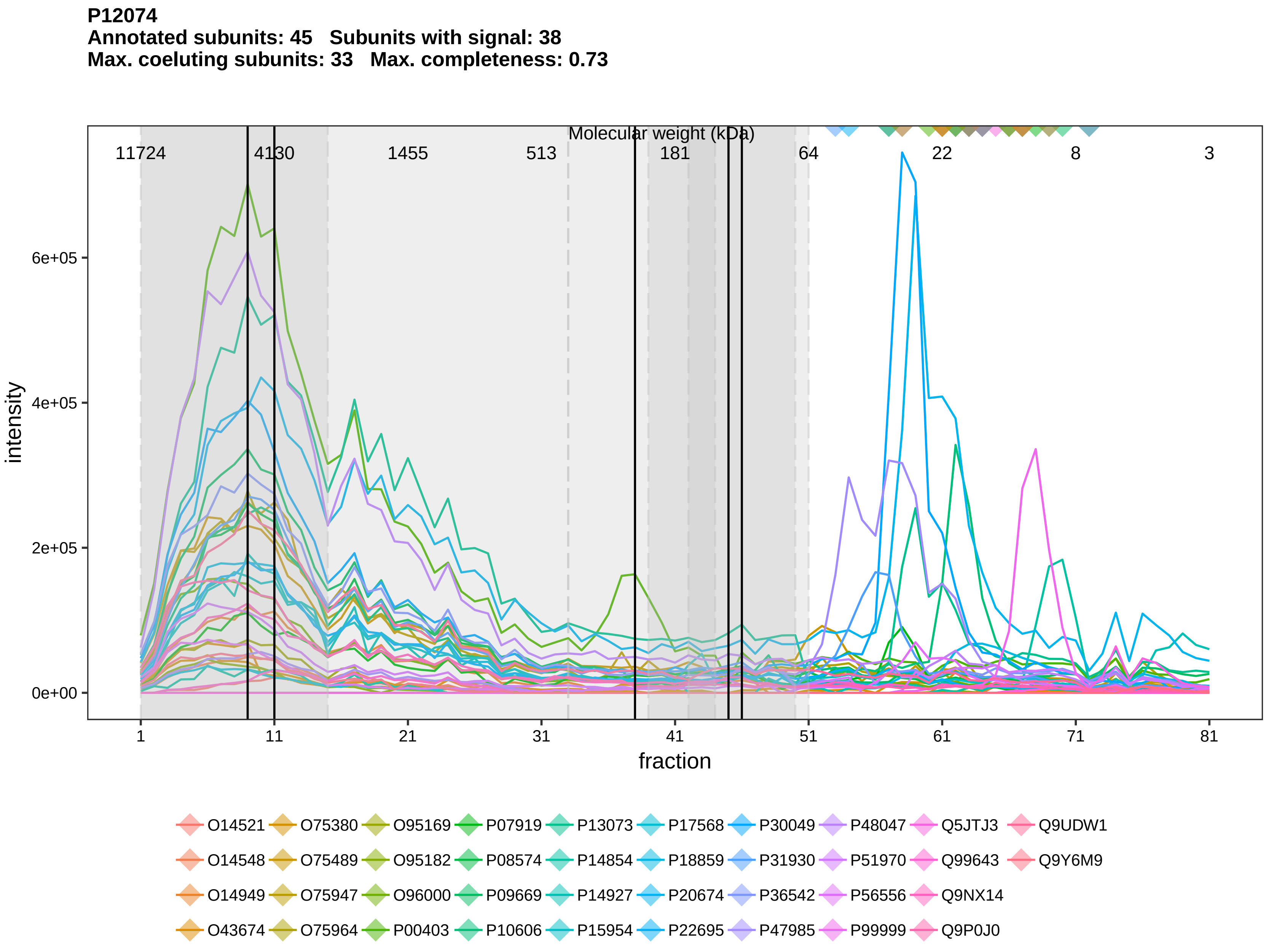
P12074
Annotated subunits: 45 Subunits with signal: 38
Max. coeluting subunits: 33 Max. completeness: 0.73
Molecular weight (kDa)
11724
4130
1455
513
181
64
22
8
3
intensity
6e+05
4e+05
2e+05
0e+00
fraction
1
11
21
31
41
51
61
71
81
O14521 O75380 O95169 P07919 P13073 P17568 P30049 P48047 Q5JTJ3 Q9UDW1
O14548 O75489 O95182 P08574 P14854 P18859 P31930 P51970 Q99643 Q9Y6M9
O14949 O75947 O96000 P09669 P14927 P20674 P36542 P56556 Q9NX14
O43674 O75964 P00403 P10606 P15954 P22695 P47985 P99999 Q9P0J0
